# Evaluating the Dental Hygienists' Exposure to the Risk of Musculoskeletal Disorders

**DOI:** 10.1055/s-0042-1750772

**Published:** 2022-09-08

**Authors:** Waldemar Ćwirzeń, Leopold Wagner

**Affiliations:** 1 Department of Dental Propaedeutics and Prophylaxis, Medical University of Warsaw, Warsaw, Poland

**Keywords:** body position, dental hygienist, musculoskeletal disorder risk, overloads, static load

## Abstract

**Objectives** The aim of the study was to evaluate dental hygienists' exposure to the risk of musculoskeletal disorders (MSDs), by the worksheets compatible with the Rapid Entire Body Assessment (REBA).

**Materials and Methods** The research included 272 dental hygienists aged 23 to 52 years from the two administrative regions of Poland.

**Statistical Analysis** STATISTICA 12 and Microsoft Excel were used to analyse the results. The level of significance was *p* <0.05. The normality of the distribution was tested with the Shapiro–Wilk test. The relationships between the variables were assessed with the *χ* 2 test. Due to the lack of “normality” of the REBA risk distribution, the Mann–Whitney test was used to verify the hypotheses.

**Results** Overall, 48.5% of the examined showed a neck flexion >20, torsion of 80.1%, and 37.5% declared the presence of both types of loads. Also, 14.3% of the examined kept a vertical position, 53.7% flexion to the torso up to 20degrees, 31.4% to 60degrees, and 2.1% to >60degrees. Further, 78.3% of people indicated that they twist the torso. Then, 7% marked the low load's arms position, 45.6% marked the range from 20 to 45degrees, and 39.7% marked the range from 45 to 90degrees. Over 55% showed additional load related to the raising or abduction of the shoulders. Overall, 43% showed a wrong position of forearms. Also, 62.9% showed wrists flexed <15° and the rest showed >15degrees. Again, 79% showed additional twisting and flexion of the wrists. Almost 75% of the examined are exposed to overloads associated with the static load. The examined are not exposed to excessive loads resulting from sudden exertion. REBA scores indicate that the negligible MSDs risk concerns 0.7% examined; low risk, 5.5%; medium risk, 33,1%; high risk, 49.3%; and very high risk, 11.4%. The correlation coeffcients analysis showed that exposure risk is strongly correlated with the overloads on the tested parts of the body in both groups.

**Conclusion** The levels of MSDs risk indicate that hygienists more often should be subjected to periodic check-ups in the workplace. They also need ergonomic interventions (education, preventive technique, physical activity, and improvement of the working environment) and modifications of hygienist's college programs in the field of work ergonomics can be also considered.

## Introduction

Musculoskeletal disorders (MSDs) are one of the important problems occurring in the dental team. 1 It was found that in the dentistry environment, the ergonomic risk reaches 87.5%. 2 3 This risk is related to maintaining a prolonged unsupported position, forceful exertion, precise and/or repetitive motions, mental workload, inadequate lighting, exposure to vibrations, and using thin instruments. 4 5 Taking a nonergonomic position already happens during studies. 1 6 7 8 9 Even in a proper sitting position, more than 50% of the muscles contract can lead to the appearance of microtrauma in bones, joints, muscles, ligaments, nerves, and blood vessels. 5 7 8 Lack of knowledge or the habitual taking of a bad position, make not everyone sees this problem. According to a performed meta-analysis, it was found that MSDs occur in 10 .8 to 97.9% of dental professionals. 10

MSDs are common among dental hygienists because more than one-third of them require analgetics or treatment for complications caused by an overload. 11 In the United States, absenteeism by hygienists due to MSDs is on average 5 weeks per year, and some of them reduce their working time or even change jobs. 6 12 Similarly, in European countries, MSDs are one of the main reasons for absenteeism and include 39% of absences of more than 2 weeks. 13 So, MSDs lead to premature leaving of the workplace and are associated with high costs due to absenteeism, limitation of efficiency, increasing the need for treatment and rehabilitation, and workers' compensation. 14

One of the tools for the assessment of posture workplace is the Rapid Entire Body Assessment (REBA) which identifies the forced positioning of different parts of the body and for determining the level of exposure to MSDS. 15

The aim of the study was to evaluate dental hygienists' exposure to the risk of MSDs, by the worksheets compatible with REBA.

## Materials and Methods

The research included 272 dental hygienists aged 23 to 52 years from the two administrative regions of Poland. The study was performed in two stages, at the 1-year interval. Group 1 included 188 of 800 professionally active people and the second 84 of 500. Inclusion criteria were work in general dentistry practice, four-hand work, performing the same procedures, and acceptance of the survey study. Exclusion criteria were participation in specific procedures, symptoms of MSDs, rehabilitation, use of nonsteroidal anti-inflammatory drugs (NSAIDs), and refusal to participate in the study. The work experience of the respondents ranged from 5 to ≥20 years. Also, 12% of the examined claim that their weekly working time is 40 to 60hours, 30 to 40hours for 64%, 20hours for 21%, and 15hours for 3%. The study was performed using worksheets compatible with REBA. Prior to the study, the agreement of the respondents and their employers was obtained and informed to the University Bioethical Commission. For the purposes of this study, each of the participants also had to sign an agreement to the processing of personal data in accordance with the General Data Protection Regulation. 16 In the worksheet, participants marked drawings imitating the examined body parts positions taken during work, specified force/load when lifting and/or moving additional weight, quality of the handhold, and a static load/activity during the work. Assessment of the position of the arm, forearm, wrist, and the quality of the handhold was performed separately for the right and left hands. The points with each criterion were summed up to determine the overall REBA scores and the level of risk to MSDs on a 5-point scale, from negligible to very high. 15 Both groups were also compared in terms of differences in the structure of the distribution of the “risk” parameter.

The STATISTICA 12 (StatSoft Poland) analytics tool and Microsoft Excel 2016 were used for the statistical analysis of the results. The level of significance was *p* <0.05. The normality of the distribution was tested with the Shapiro–Wilk test. The relationships between the variables were assessed with the *χ* ^2^ test. Due to the lack of “normality” of the REBA risk distribution, the Mann–Whitney test was used to verify the hypotheses. To determine the relationships between the ordinal variables, the gamma correlation coefficient was calculated. It also investigated which outcomes were most closely related to REBA risk, and which outcomes were correlated with each other.

## Results

The obtained results are presented in the five tables.

The results of the taken position of the neck, torso, and legs and subjective determination of force/load when lifting and/or moving additional weight and static load/activity during the work taken in both examined groups showed in Table 1 . Each criterion has an assigned number of points.

**Table 1:** Results of the neck, torso, and legs position and subjective determination of force/load when lifting and/or moving additional weight and static load/activity during the work, group I—188 persons and group II—84

Criteria		Point	Group I (%)	Group II (%)
Body parts	Neck flexion less than 20degrees	+1	53.7	46.4
	Neck flexion more than 20degrees	+2	46.3	53.6
	Neck twisted	+1	78.7	83.3
	Neck straight	+1	21.3	16.7
	Presence of two risks (neck flexion >20degrees and twisting)	+3	36.2	40.5
	Torso straight	+1	3.7	38.1
	Torso flexion less than 20degrees	+2	62.8	33.3
	Torso flexion 20 to 60degrees	+3	31.4	21.4
	Torso flexion more than 60degrees	+4	2.1	7.1
	Torso twisted	+1	79.3	76.2
	Equal load of legs	+1	27.1	13.1
	Angle setting the knee joint 30 to 60degrees	+1	61.2	66.7
	Angle setting the knee joint more than 60degrees	+2	11.7	20.2
Lifting/moving additional weight	Load with a mas less than 5kg	0	50.0	57.1
	Load with a mas 5 to 10kg	+1	47.3	35.7
	Load with a mas more than 10kg	+2	2.7	7.1
	Quick lifting	+1	0.0	
Static load/activity during the work	Static effort over 1minute	+1	10.1	38.1
	Activity repeated four times per minute or more often	+1	81.4	58.3
	Large changes in body posture or unstable position	+1	7.4	3.6

Note: The “Point” column indicates the number of points added for that evaluation criterion.

The results of the take position of the arms, forearms, and wrists in both examined groups showed in Table 2 . Each criterion has an assigned number of points.

**Table 2:** Results of the arms, forearms, and wrists position in both examined groups, LH (left hand) and RH (right hand), group I—188 persons and group II—84

Criteria	Point	Group I (%)		Group II (%)	
		LH	RH	LH	RH
Arm is supported	-1	0.0	0.53	25.0	23.8
Movement arm in the sagittal plane less than 20degrees	+1	8.5	9.0	6.0	2.4
Movement arm in the sagittal plane 20 to 45degrees	+2	48.4	49.5	26.2	36.9
Movement arm in the sagittal plane 45 to 90degrees	+3	43.1	41.0	42.9	36.9
The arm is angled at 90degrees	+4	0.0			
Raised/abducted of the arm	+1	69.7	79.8	58.3	52.4
Forearms raised at an angle in the range of 60 to 100degrees	+1	56.9	57.4	52.4	59.6
Forearms raised at an angle from 0 to 60 or above 100degrees	+2	43.1	42.6	47.6	40.5
Wrist flexion 0–15degrees	+1	56.4	62.8	71.4	69.0
Wrist flexion more than 15degrees	+2	43.6	37.2	28.6	31.0
Wrist no flexion from the midline and/or twisted	+1	16.0	16.0	39.3	32.1
Wrist lateral flexion and/or twisted	+1	84.0	84.0	60.7	67.9

Note: The “Point” column indicates the number of points added or subtracted for that evaluation criterion.

The results of the quality grip on a scale of 0 to 3 in the left and right hands in both groups showed in Table 3 .

**Table 3:** Results of the quality handhold on a scale of 0–3, LH (left hand) and RH (right hand), group I—188 persons and group II—84

Criteria	Point	Group I (%)		Group II (%)	
		LH	RH	LH	RH
Good	0	66.5	78.7	47.6	75.0
Acceptable	1	29.8	19.2	45.3	21.4
Poor	2	3.7	2.1	7.1	3.6
Unacceptable	3	0.0			

The overall REBA score and the level of risk of MSDs in both groups showed in Table 4 .

**Table 4:** Overall REBA score and level of exposure to MSDs, group I—188 persons and group II—84

MSDs risk	Score	Group I (%)	Group I (%)I
Negligible	1	0.0	2.4
Low	2–3	0.6	16.6
Medium	4–7	30.3	39.3
High	8–10	58.5	28.6
Very high	11–15	10.6	13.1

Abbreviations: MSDs, musculoskeletal disorders; REBA, the Rapid Entire Body Assessment.

Table 5 shows the Mann–Whitney *U* -test for the variable group. Statistical significance set at the level of *p* <0.05000.

**Table 5:** U Mann–Whitney test for relative to the variable groups, N-important: group I—188 and group II—84

Parameters	Sum of ranks (group 1)	Sum of ranks (group 2)	*U* -statistic	*Z* -statistic	*p* -Value
Neck	26,266.50	10,861.50	7,291.50	1.097507	0.272421
Torso	28,063.00	9,065.00	5,495.00	**4.233862**	**0.000023**
Legs	23,928.50	13,199.50	6,162.50	**−3.19266**	**0.001410**
Arm R	8,081.50	29,046.50	4,511.50	**−5.86352**	**0.000000**
Arm L	9,413.00	27,715.00	5,843.00	**−3.54240**	**0.000397**
Force/load	26,052.00	11,076.00	7,506.00	0650662	0.515265
REBA scores	8,747.50	28,380.50	5,177.50	**−4.58725**	**0.000004**

Abbreviations: L, left; R, right; REBA, the Rapid Entire Body Assessment.

Note: Statistical significance at the level of *p* <0.05000 and highlighted in bold.

In groups I and II, a very high correlation associated the risk of REBA with the load on the neck ( *γ* =0.66 and 0. 71, respectively) and torso ( *γ* =0.79 and 0.81, respectively).

The strength of the correlation of neck loads in relation to the torso in group I was medium and amounted to *γ* =0.30 and in group II, it is high at *γ* =0.61.

There is a high correlation between the risk of REBA and the load on the legs, in both groups, *γ* =0.56.

In group, I the correlation between arms workload and risk of REBA was medium, right arm: *γ* =0.38 and left arm: *γ* =0.41. In group II, armloads were better correlated with the risk of REBA, the right *γ* =0.53 and left *γ* =0.55.

In group, I the correlation between the load on the left arm and torso has a low force ( *γ* =0.16) and in group II medium ( *γ* =0.31). The correlation between torso and legs in groups I and II was *γ* =0.39 and 0.32, respectively. Whereas, the right arm was a strong correlation ( *γ* =0.65) with the load on the torso in group II which is a significant difference compared with group I. In group II, there occurred an average correlation between the load on the right arm and the neck, *γ* =0.44 and torso and legs, *γ* =0.29, whereas in group I, there was no such correlation.

## Discussion

Results of the present study demonstrated a relatively high exposure of the studied groups of hygienists to the appearance of MSDs. Average REBA scores indicate that the negligible MSDs risk concerns 0.7% examined; low risk, 5.5%; medium risk, 33.1%; high risk, 49.3%; and very high risk, 11.4%. The analysis of correlation coefficients showed that in both groups, the risk of exposure according to REBA is strongly correlated with the overloads on the tested parts of the body.

REBA risk in groups I and II differs significantly (Mann–Whitney test) and is higher in group I than in group II and differs in structures.

Furthermore, 48.5% of the examined showed a neck flexion of >20, 80.1% of torsion, and 37.5% declared the presence of both types of loads. Only14.3% of the examined maintained a vertical position, 53.7% showed a bent torso up to 20degrees, 28.3% showed up to 60degrees, and 3.7% showed more than 60degrees. Also, 78.3% of hygienists indicated that they twist their torso during work; 62.9% examined showed bend knees from 30 to 60degrees and 14.3% showed above 60degrees. Again, 7% of the examined raised their arms below 20degrees, 45.6% raised in the range from 20 to 45degrees, 39,7% from 45 to 90degrees, and only 7.7% performed work with supporting arms. Also, over 55% of hygienists showed additional load related to the raising or abduction of the shoulders. Overall, 43% examined showed the wrong position of forearms and the rest only slight loads. Then, 62.9% of the hygienists showed wrists flexed <15degrees and the rest showed over 15degrees. Finally, 79% of those examined showed additional twisting and flexion of the wrists to the side and 75% of the examined showed overloads were associated with the static load.

Moreover, 41.5% of the examined were shown to have loads associated with lifting additional weight from 5 to 10kg and 5% were shown a load of >10kg. Nobody marked the quick lifting of the weight. There were no significant differences in the subjective determination of load when lifting and/or moving additional weight between the studied groups (Mann–Whitney test). Based on the analysis of the results, it can be concluded that examined are not exposed to excessive loads resulting from sudden exertion.

In the case of the right hand, the good hold was found in 78.7% of those examined, acceptable in 19.3%, and poor in 2%. In the case of the left hand, a good hold was observed in 66.5% of examined, an acceptable in 29.8%, and poor in 3.7%. Unacceptable handle not detected. In the first group, there were significant differences in the quality of holding with the left hand (Mann–Whitney test). On the right hand, there were no significant differences.

These obtained results are in accordance with several studies. In the case of the cervical region of the spine, Morse et all showed that improper bending of the neck concerns up to 96% of dental hygienists and approximately 89% of hygiene students. 8 In another study authors observed that 90% of the work time, hygienist hold their heads from 17 to 39degrees and 10% greater than 40degrees. 17 It was found that pain in the neck area is reported by 54 to 85% of hygienists. 11 18 The last research conducted in Germany showed that within 12 months neck and back pain was reported by approximately 85.6% of dentists and dental students. 9

Based on the research, it was found that 68% of hygienist has lower back pain, 11 30% report moderate/severe pain in the lower/upper back, and 18% pain in the hips. 19 Also, 52% of hygienists report MSDs on the upper and lower back. 18

Pain localized in the legs affects 8.3% of American hygienists, 20 and in Sweden, 23% of hygienists complain of pain in the hips, thighs, and knees. 21

Shoulder problems occur in 35 to 76% of hygienists 8 11 and found a relationship between pain in the shoulder joint area and forced body position during work. 5 22 Overall, 60% of U.S. hygienists and 81% of Swedish reported a higher complaint of shoulder pain. 23 In another study, 35% of dental hygienists report moderate or severe pain in the shoulders. 19

Also, 69.5% of Australian dental hygienists reported pain in the hand due to overload in this region. 11 Wrist pains can also be related to the type of performed procedures. According to studies pain in the wrist is correlated with the performance of supra- and subgingival scaling. 5 24 This situation applies not only to professionals but also to students. Forearm and wrist problems cause dental hygienists to seek medical help and are one of the main reasons for their absenteeism. 5 25 In this professional group, carpal tunnel syndrome is a frequent occurrence, for example, 44.2% of American hygienists have visible symptoms of this syndrome, 26 and hand pain of Swedish hygienists is also associated with it. 27 According to the Bureau of Labor Statistics (2002), dental hygienist ranks first among all professions in the prevalence of carpal tunnel syndrome per 1,000 employees.

The results concerning static loads obtained in the present study are at a higher level than those described by other authors, for example, according to Morse et al, 63% of dental hygienists keep a static position. 8

The reason for the presence of those disturb is related to long-term overload of the musculoskeletal system due to awkward position, performing repetitive movements and using thin and vibrating tools. Lack of adequate reaction and/or long-term use of NSAIDs can lead to biomechanical disorders. 28 29 The neck area is the most likely to develop MSDs because of the frequent head tilts >20degrees and twisting of the neck during dental procedures. Such a position disturbs the muscular balance (the neck extensors are strongly tense because they must counteract the force of gravity), and the joints of the spine are overloaded. 28 30 Prolonged maintenance of this position promotes the loss of cervical lordosis, disc protrusion, functional disorders, and pain. Maintaining a stable head position also requires the participation of upper thoracic spine muscles which also contract constantly. The load on the cervical spine is not gendered specific. 31

The load on the shoulder area is related to long-term work with shoulder abduction of >45degrees, repetitive activities, and vibrations. The result of the forced position is a contraction of the deltoid, supraspinatus trapezius, and serratus anterior muscles which leads to their overload and fatigue. According to the available data, women are more exposed to this type of load. 31

MSDs in the lumbar area are associated with the loss of lumbar lordosis. Maintaining an incorrect sitting position with a forward lean, without tilting the hips, leads to the weakening of the stabilizing muscles of the lumbar spine. The load on the lumbar region is not gendered specific. 31

The appearance of MSDs in the dorsal area is associated with the loss of cervical and lumbar lordosis, leaning the head forward, and prolonged work in a sitting position. According to the available data, women are more exposed to this type of load. 31

Long-term work with overloaded legs not only causes pain in muscles, ligaments, and bones but also promotes the expansion of the leg veins and flat feet. 17 22

The use of thin instruments or micromotor handpieces or ultrasonic devices is conducive to the formation of an uncomfortable finger system and fatigue of some muscle groups in the hands. This manifests as numbness, tingling, stiffness, paresthesia, contractures, and hand pain and may lead to tenosynovitis or Dupuytren's contracture. 24

Exposure to long-term vibrations is conducive to the emergence of many nonspecific symptoms. Changes in the vascular, nervous, and osteoarticular systems commonly known as vibration syndrome can occur. This disease initially manifests itself in the form of spasm which is associated with pain and leads to changes in the wrist joints. Numbness in the hands, tingling sensation, and loss of strength may be also associated when vibrations are transferred to the hand. 32

The level of MSDs risk depends also on the type of dental procedure and is signiﬁcantly greater in the case of oral surgery and general dentistry than in endodontology or orthodontics. 33 A recent study performed in Canada showed that 83% of dental hygienists performing oral hygiene procedures reported symptoms of MSDs. 34 When the hygienist concentrates on the realization of the procedure, it may ignore signs of fatigue. A prolonged lack of rest leads to disorders of blood circulation and metabolism in the muscles which can damage their cells.

The severe acute respiratory syndrome-coronavirus-2 (SARS-COV-2) pandemic additionally increased the exposure of members of the dental team to workload. 35 36 37 The need to use personal protective equipment such as N95 (FFP2/3) masks, safety uniforms, impervious disposable gown with head cap, glasses, goggles, polycarbonate shield, etc., 36 reduces the comfort of the work environment (e.g., deterioration of vision of the work area, sweating, and possible breathing difficulties), makes it difficult to perform procedures (restrictions on movement and forced work position), and increases the level of mental workload (stress). 37

The clinical practice involves several wrong positions and nonergonomic postures. This is due to the poor preparation of the dental office, wrong positioning of the patient, no full match with the operator, and lack of knowledge or habits.

Bad preparation of the dental office causes the hygienist when reaching for instruments or materials, to twist her header, twist and tilt her torso, and pull her arms forward.

The lack of a full fit when working with four hands causes the hygienist to tilt the head, twist the neck, twist and bend the back, and raises the hands. The necessity to change the operator's instruments can force the hygienist to break the support points and perform the nonrecommended movements. When sitting at the same height as the operator, the hygienist must tilt the torso to reach the treatment area.

Positioning the patient too high forces the arms to be raised and the shoulder muscles to tense, while the too low positioning causes the back to bend and load the sacral part of the spine. Too little tilt of the patient's head promotes shoulder abduction.

The tendency to connect the knees and perpendicular position to the dental chair moves the hygienist away from the patient and forces her to lean toward the treatment field and bend their back. A similar situation occurs when the hygienist is placed too close to the operator. Working independently at 10.00 or 11.00 forces a one-sided tilt and lifts the arms to the side which additionally puts loads on the shoulder.

The level of exposure to MSDs indicates the need for ergonomic interventions in dental hygienists. The recommended solutions are as follows:

- Ergonomic education (e.g., dedicated courses, training, shows, and others).
- Preventive techniques (e.g., adopting a neutral position, dynamising the position during work, and introducing microbreaks in work).
- Physical activity (relaxation gymnastics, preventive and stretching exercises, fitness, yoga, kinesiotherapy, and meditation).
- Modification of work position (e.g., use of an ergonomic stool, sitting slightly higher than the dentist and at an angle to the patient, hips higher than the knees, back straight, and working independently at 12.00).
- Improvement/modification of the working environment by the employer (e.g., changing the equipment and/or arrangement, compliance with working time, and introduction of regular breaks).

It is assumed that the acceptable REBA level in the workplace should be below 4 and the goal is to obtain the REBA index at level 1 or even lower.

## Conclusion

The levels of MSDs risk indicates that hygienists more often should be subject to periodic check-ups in the workplace. This professional group should be also implemented corrective and preventive actions and periodic ergonomics training. Modifications of dental hygienist's college programs in the field of work ergonomics can also be considered.

## References

[1] Ng A Hayes M J Polster A Musculoskeletal disorders and working posture among dental and oral health students Healthcare (Basel) 2016 4 01 13 27417601 10.3390/healthcare4010013 PMC4934547

[2] De Sio S Traversini V Rinaldo F Ergonomic risk and preventive measures of musculoskeletal disorders in the dentistry environment: an umbrella review PeerJ 2018 6 e4154 29362689 10.7717/peerj.4154 PMC5772380

[3] Ísper Garbin A J Barreto Soares G Moreira Arcieri R Adas Saliba Garbin C Siqueira C E Musculoskeletal disorders and perception of working conditions: a survey of Brazilian dentists in São Paulo Int J Occup Med Environ Health 2017 30 03 367 377 28481371 10.13075/ijomeh.1896.00724

[4] Barry R M Spolarich A E Weber M Krause D Woodall W D Bailey J H Impact of Operator positioning on musculoskeletal disorders and work habits among Mississippi dental hygienists J Dent Hyg 2017 91 06 6 14 29378801

[5] Hayes M J Taylor J A Smith D R Predictors of work-related musculoskeletal disorders among dental hygienists Int J Dent Hyg 2012 10 04 265 269 22081978 10.1111/j.1601-5037.2011.00536.x

[6] Warren N Causes of musculoskeletal disorders in dental hygienists and dental hygiene students: a study of combined biomechanical and psychosocial risk factors Work 2010 35 04 441 454 20448323 10.3233/WOR-2010-0981

[7] Hayes M J Smith D R Taylor J A Musculoskeletal disorders in a 3 year longitudinal cohort of dental hygiene students J Dent Hyg 2014 88 01 36 41 24563051

[8] Morse T Bruneau H Michalak-Turcotte C Musculoskeletal disorders of the neck and shoulder in dental hygienists and dental hygiene students J Dent Hyg 2007 81 01 10 17362608

[9] Ohlendorf D Naser A Haas Y Prevalence of musculoskeletal disorders among dentists and dental students in Germany Int J Environ Res Public Health 2020 17 23 8740 33255491 10.3390/ijerph17238740 PMC7727829

[10] Lietz J Kozak A Nienhaus A Prevalence and occupational risk factors of musculoskeletal diseases and pain among dental professionals in Western countries: a systematic literature review and meta-analysis PLoS One 2018 13 12 e0208628 30562387 10.1371/journal.pone.0208628 PMC6298693

[11] Hayes M J Smith D R Taylor J A Musculoskeletal disorders and symptom severity among Australian dental hygienists BMC Res Notes 2013 6 250 23822098 10.1186/1756-0500-6-250 PMC3704656

[12] Garbin A J Garbin C A Moimaz S A Baldan R C Zina L G Dental practice and musculoskeletal disorders association: a look at the evidence Arch Environ Occup Health 2011 66 01 26 33 21337183 10.1080/19338244.2010.506493

[13] Oberg T Oberg U Musculoskeletal complaints in dental hygiene: a survey study from a Swedish county J Dent Hyg 1993 67 05 257 261 8270993

[14] Worker health chartbook 2004Accessed May 18, 2022 at:https://www.cdc.gov/niosh/docs/2004-146/

[15] Hignett S McAtamney L Rapid entire body assessment (REBA) Appl Ergon 2000 31 02 201 205 10711982 10.1016/s0003-6870(99)00039-3

[16] Regulation (EU) 2016/679 of the European Parliament and of the Council of 27 April 2016 on the protection of natural persons with regard to the processing of personal data and on the free movement of such data, and repealing Directive 95/46/EC (General Data Protection Regulation)Accessed May 18, 2022 at:https://www.eumonitor.eu/9353000/1/j9vvik7m1c3gyxp/vk3sygzz13zi

[17] Branson B G Black M A Simmer-Beck M Changes in posture: a case study of a dental hygienist's use of magnification loupes Work 2010 35 04 467 476 20448325 10.3233/WOR-2010-0983

[18] Netanely S Luria S Langer D Musculoskeletal disorders among dental hygienist and students of dental hygiene Int J Dent Hyg 2020 18 02 210 216 32012436 10.1111/idh.12428

[19] Humann P Rowe D J Relationship of musculoskeletal disorder pain to patterns of clinical care in california dental hygienists J Dent Hyg 2015 89 05 305 312 26519494

[20] Lalumandier J A McPhee S D Parrott C B Vendemia M Musculoskeletal pain: prevalence, prevention, and differences among dental office personnel Gen Dent 2001 49 02 160 166 12004695

[21] Akesson I Johnsson B Rylander L Moritz U Skerfving S Musculoskeletal disorders among female dental personnel–clinical examination and a 5-year follow-up study of symptoms Int Arch Occup Environ Health 1999 72 06 395 403 10473839 10.1007/s004200050391

[22] Morse T F Michalak-Turcotte C Atwood-Sanders M A pilot study of hand and arm musculoskeletal disorders in dental hygiene students J Dent Hyg 2003 77 03 173 179 14596163

[23] Hayes M Cockrell D Smith D R A systematic review of musculoskeletal disorders among dental professionals Int J Dent Hyg 2009 7 03 159 165 19659711 10.1111/j.1601-5037.2009.00395.x

[24] Dong H Loomer P Barr A Laroche C Young E Rempel D The effect of tool handle shape on hand muscle load and pinch force in a simulated dental scaling task Appl Ergon 2007 38 05 525 531 17156742 10.1016/j.apergo.2006.09.002 PMC1974884

[25] Howarth S J Grondin D E La Delfa N J Cox J Potvin J R Working position influences the biomechanical demands on the lower back during dental hygiene Ergonomics 2016 59 04 545 555 26230089 10.1080/00140139.2015.1077274

[26] Anton D Rosecrance J Merlino L Cook T Prevalence of musculoskeletal symptoms and carpal tunnel syndrome among dental hygienists Am J Ind Med 2002 42 03 248 257 12210693 10.1002/ajim.10110

[27] Åkesson I Balogh I Hansson GÅ Physical workload in neck, shoulders and wrists/hands in dental hygienists during a work-day Appl Ergon 2012 43 04 803 811 22208356 10.1016/j.apergo.2011.12.001

[28] Palmer K T Smedley J Work relatedness of chronic neck pain with physical findings–a systematic review Scand J Work Environ Health 2007 33 03 165 191 17572827 10.5271/sjweh.1134

[29] Kalluri A Puranik M P Uma S R Musculoskeletal disorders in dental workplace: a comprehensive review Int J Appl Dent Sci 2018 4 140 145

[30] Bone and Joint Decade 2000-2010 Task Force on Neck Pain and Its Associated Disorders Côté P van der Velde G Cassidy J D The burden and determinants of neck pain in workers: results of the Bone and Joint Decade 2000-2010 Task Force on Neck Pain and Its Associated Disorders Spine 2008 33(Suppl 4)S60 S74 18204402 10.1097/BRS.0b013e3181643ee4

[31] Gandolfi M G Zamparini F Spinelli A Risi A Prati C Musculoskeletal disorders among Italian dentists and dental hygienists Int J Environ Res Public Health 2021 18 05 2705 33800193 10.3390/ijerph18052705 PMC7967428

[32] Rytkönen E Sorainen E Leino-Arjas P Solovieva S Hand-arm vibration exposure of dentists Int Arch Occup Environ Health 2006 79 06 521 527 16421714 10.1007/s00420-005-0079-y

[33] Holzgreve F Fraeulin L Betz W A RULA-based comparison of the ergonomic risk of typical working procedures for dentists and dental assistants of general dentistry, endodontology, oral and maxillofacial surgery, and orthodontics Sensors (Basel) 2022 22 03 805 35161550 10.3390/s22030805 PMC8839213

[34] Harris M L Sentner S M Doucette H J Brillant M GS Musculoskeletal disorders among dental hygienists in Canada Can J Dent Hyg 2020 54 02 61 67 33240365 PMC7668274

[35] Sharma S Parolia A Kanagasingam S A review on COVID-19 mediated impacts and risk mitigation strategies for dental health professionals Eur J Dent 2020 14(S 01):S159 S164 33167046 10.1055/s-0040-1718240 PMC7775253

[36] Gandolfi M G Zamparini F Spinelli A Sambri V Prati C Risks of aerosol contamination in dental procedures during the second wave of COVID-19-experience and proposals of innovative IPC in dental practice Int J Environ Res Public Health 2020 17 23 8954 10.3390/ijerph17238954 33271981 PMC7729834

[37] Du M Hu K Frontline health care workers' mental workload during the COVID-19 pandemic: a cross-sectional study Asia Pac J Public Health 2021 33(2,3):303 305 33622039 10.1177/1010539521997257

